# Outcome of severe COVID-19: spotlight on fatigue, fatigability, multidomain complaints and pattern of cognitive deficits in a case series without prior brain dysfunction and without COVID-19-related stroke and/or cardiac arrest

**DOI:** 10.1186/s13256-023-04300-6

**Published:** 2024-02-02

**Authors:** Valérie Beaud, Sonia Crottaz-Herbette, Vincent Dunet, Jean-François Knebel, Pierre-Alexandre Bart, Stephanie Clarke

**Affiliations:** 1 Service of Neuropsychology and Neurorehabilitation, Lausanne University Hospital and University of Lausanne, Av. Pierre-Decker 5, 1011 Lausanne, Switzerland; 2 Service of Diagnostic and Interventional Radiology, Lausanne University Hospital and University of Lausanne, 1011 Lausanne, Switzerland; 3 Service of Internal Medicine, Lausanne University Hospital and University of Lausanne, 1011 Lausanne, Switzerland

**Keywords:** Severe COVID-19, Fatigue, Fatigability, Mental effort, Multidomain complaints, Cognitive impairment, Everyday living impact

## Abstract

**Background:**

Population-wide surveys and large-scale investigations highlighted the presence of cognitive deficits in the acute and postacute stages of severe COVID-19; a few studies documented their occurrence in cases without prior or COVID-19-related brain damage. The evolution of cognitive deficits in the latter population and their relationship to the post-COVID-19 fatigue syndrome are poorly understood.

**Case presentation:**

We report the outcome at 12 months after severe COVID-19 involving an intensive care unit stay and mechanical ventilation in six (five Caucasian and one Asian) patients (age range: 53–71 years, mean age 61.7 ± 6.5 years) without history of prior brain dysfunction and without stroke and/or cardiac arrest during or after COVID-19. All patients reported pervading mental and physical fatigue as well as numerous multidomain complaints, which impacted everyday life. Individual patients described mental fatigability, apathy, and/or anxiety. Standardized neuropsychological tests revealed isolated symptoms of cognitive dysfunction or performance at the lower limit of the norm in the attentional, executive, and/or working memory domains in four of the six patients. Somatic scales documented dyspnoea, muscle weakness, olfactory disorder, and/or minor sleep problems in some, but not all, patients.

**Conclusion:**

Fatigue, fatigability, multidomain complaints, cognitive difficulties, or dysfunction, as well as isolated neurobehavioral and/or psychiatric and/or somatic symptoms, tend to occur in the aftermath of severe COVID-19 and persist at 12 months, even in the absence of prior and/or COVID-19-related brain damage. This clinical situation, which impacts everyday life, calls for a detailed investigation of patients’ complaints, its neural underpinning, and an elaboration of specific rehabilitation programs.

## Introduction

Cognitive deficits were often reported during the acute and postacute stages of severe acute respiratory syndrome coronavirus 2 (SARS-CoV-2) infection [1–3]. Early reports from acute wards described, in addition to other neurological symptoms, the occurrence of impaired consciousness, which tended to be more frequent in association with severe, rather than nonsevere, pneumonia [4]. A large proportion of patients who suffered from acute respiratory distress syndrome (ARDS) and necessitated mechanical ventilation presented agitation and confusion (69%) and/or dysexecutive syndrome (36%) after the stop of sedation [5]. During the acute stage, the presence of neurological signs, including cognitive impairment, was shown to be associated in some, but not all patients with neuroradiological findings such as microbleeds, large parenchymal and subarachnoidal hemorrhage, acute or subacute stroke, watershed white matter hyperintensities, and/or acute disseminated encephalomyelitis [6].Coronavirus disease 2019 (COVID-19)-related brain damage was also demonstrated in postmortem magnetic resonance imaging (MRI) and in autopsies, with the most common findings being neuroinflammatory changes [7].

Patients, who had no history of prior brain damage or dysfunction and who did not sustain radiologically detectable brain damage during COVID-19, were reported to suffer from cognitive dysfunction during the postcritical acute stage: moderate-to-severe cognitive deficits were found in 38% of patients, mild in 31% of patients, and none in 31% of patients [8]. Postacute outcome has been evaluated in several studies, both at the admission to inpatient rehabilitation units or upon discharge home. There is concurring evidence that patients often present cognitive deficits at the beginning of inpatient rehabilitation, mostly impacting working memory, executive functions, divided attention, and processing speed [9–12]. Full-blown cognitive deficits tend to be less frequent in patients who were discharged home after the acute stage, although complaints of memory and concentration dysfunction as well as of fatigue are often reported [13–16].

A large-scale cross-sectional online study reported that patients, who recovered from COVID-19, presented cognitive sequelae, in particular, if the severity of the disease required hospitalization; the findings of this study emphasized a multidomain impact on cognition [17]. The presence of severe neurological and/or psychiatric symptoms, which affect patients who suffered from COVID-19, was revealed with the help of the online case report database of the Association of British Neurologists, the British Association of Stroke Physicians, and the Royal College of Psychiatrists [18]. Large-scale analysis of insurance health records confirmed substantial neurological and psychiatric morbidity 6 months after COVID-19 [19]. These findings call for further investigations into the nature, severity, and evolution of cognitive, behavioral, and psychiatric deficits.

Three issues are currently of great interest. First, very little is known about the extent of cognitive recovery, the profile of putative sequelae, and their impact on everyday living. Data from follow-up of patients who suffered from infections with other coronaviruses indicate that lasting sequelae are likely to occur and to have deep impact on social and professional integration [20]. Second, the relationship between the high incidence of fatigue, which has been reported in the aftermath of COVID-19 [21, 22] and the presence of cognitive deficits needs to be investigated. Third, it is unclear whether cognitive sequelae of COVID-19 represent worsening of preexisting brain dysfunction or whether they can appear de novo. Patients with prior cognitive impairment were explicitly excluded in some [16, 23] but not in other studies [11].

We provide here the missing evidence on a very specific population of intensive care unit (ICU)-survivors of severe COVID by reporting cognitive, neurobehavioral, psychiatric, somatic, and functional outcome at 12 months. All patients include in this study had severe COVID-19 involving prolonged stay in the ICU and mechanical ventilation, but had no history of prior brain damage or dysfunction. Their status was assessed with (1) standardized neuropsychological tests; (2) standardized scales of cognitive/mental and motor/physical fatigue; (3) visual analogue scales of mental fatigue/fatigability and mental effort; (4) specialized scales of neurobehavioral, psychiatric, and somatic characteristics; and (5) comparison of pre- versus post-COVID-19 complaints in cognitive, motivational, behavioral and social interactions, psychological, somatic, and functional domains. In particular, we have investigated whether (1) different measures of cognitive/mental and/or motor/physical fatigue and/or mental/cognitive fatigability provide a coherent description of our population and (2) the exacerbation of multidomain complaints is observed in addition to fatigue/fatigability. It is to be noted that perception of fatigue and fatigability were estimated in two ways, respectively: (1) subjectively with self-report scales concerning chronic characteristics (trait) of fatigue and of rest propensity, as well as momentary perceptions (state) of mental fatigue and of perceived mental effort, and (2) subjectively by quantifying the increased level of self-reported mental fatigue between two time points as well as objectively as decline in one or more aspects of performance during continuous performance of a prolonged test.

The cases reported here illustrate the lasting nature of post-COVID-19 fatigue, fatigability, multidomain complaints, and cognitive deficits, even if no radiologically detectable brain damage has been sustained. They provide thus complementary evidence to the few previously published brain damage-free cases, which were examined in the acute or early postacute stages [16, 23]. They are of high clinical relevance, emphasizing the need for detailed investigations of patient complaints and for the implementation of specific rehabilitation programs. Furthermore, they highlight the need for better understanding of the neural underpinning of the long-lasting post-COVID-19 fatigue syndrome.

## Case series presentation

Included in this study are consecutive patients, who sustained SARS-CoV-2 pneumonia between March and April 2020 (diagnosed by polymerase chain reaction) during the first COVID-19 wave and who required intubation and mechanical ventilation during their ICU stay at the Lausanne University Hospital (CHUV). Exclusion criteria comprised (1) preexisting neurocognitive impairment; (2) history of traumatic brain injury, psychiatric, oncological, or neurological disease; and/or (3) stroke or cardiac arrest as a complication of COVID-19. A total of 18 patients met the inclusion/exclusion criteria and underwent cognitive evaluation during the acute postcritical stage; 5 of the 18 patients, who were initially considered for inclusion during the acute stage, were removed from the longitudinal follow-up reported here due to a language barrier (3 patients), poor hearing (1 patient), or absence of phone contact (1 patient). Seven other patients did not wish to participate in the study. Eventually, six patients (two female and four male, mean age 61.7 ± 6.5 years) completed—in the service of Neuropsychology and Neurorehabilitation at Lausanne University Hospital between March and April 2021—the cognitive assessment at 12 months post-COVID-19 onset, which we report here (Table 1). All six patients were domiciled in the French speaking part of Switzerland. Five patients were Caucasian and one Asian. Five were married, living with their spouse, and one divorced, living alone. All patients had 12 or more years of formal education, held gainful employment, and were socially well inserted, with regular contacts with family members and friends.

**Table 1:** Patient (P1–P6) characteristics and self-reported assessment. Roman numerals represent number of years spent in education: < 12 years for I; 12 years for II; > 12 years for III. For FSMC, BFS, and ISI, bold denotes severe, bold italics denotes moderate, and italics denotes mild symptoms. For HAD, fDAS, PCL-5, ESS, sQOD-NS, QOLIBRI, and SF-36, bold denotes abnormal and italics denotes near-normal scores. F, female; M, male

	P1	P2	P3	P4	P5	P6
Age (years)	53	57	61	61	67	71
Sex (F/M)	F	M	F	M	M	M
Education level (I–III)	III	II	III	II	II	II
Duration (days) of mechanical ventilation/intensive care unit stay/acute hospitalization/postacute inpatient rehabilitation	18/21/40/53	17/21/29/14	11/12/24/0	50/67/82/37	17/21/29/31	21/31/42/52
*Fatigue scales*						
Fatigue Scale for Motor and Cognitive Functions (FSMC)						
Motor 10–50	***29***	*23*	***31***	**34**	**34**	*22*
Cognitive 10–50	***33***	*22*	***33***	***31***	***32***	*22*
Brugmann Fatigue Scale (BFS)						
Physical 0–12	***5***	0	***4***	***5***	***5***	3
Mental 0–12	***5***	2	***5***	3	***5***	2
Visual analogue scale for situational mental fatigue (VAS-SMF)						
Before cognitive testing 0–10	0	1.8	0.4	2	2.5	0.2
After cognitive testing 0–10	1.7	4.2	9.7	6.6	8.5	1.2
Visual analogue scale for perceived mental effort (VAS-PME)						
0–10	6.1	2.1	9.2	5	5.1	4.1
*Psychiatric, neurobehavioral, and somatic questionnaires and scales*						
Hospital Anxiety and Depression (HAD) scale						
Anxiety 0–21	5	6	2	1	*9*	3
Depression 0–21	6	1	2	2	1	3
French Dimensional Apathy Scale (f-DAS)						
Executive 0–24	**12**	1	**12**	1	6	0
Emotion 0–24	3	2	3	3	3	3
Initiative 0–24	9	3	8	5	8	5
DSM-5 post-traumatic stress disorder (PTSD) checklist (PCL-5)						
0–80	14	6	9	1	8	3
Epworth Sleepiness Scale (ESS)						
0–24	10	2	2	7	10	9
Insomnia Severity Index (ISI)						
0–28	*12*	5	1	3	*8*	7
Visual analogue scale for dyspnoea (VAS-D)						
0–10	0	0	3	7	7	3
Visual analogue scale for muscle weakness (VAS-MW)						
0–10	0	0	0	6	2	5
Visual analogue scale for olfactory disorders (VAS-OD)						
0–10	0	0	0	0	0	9
Short version of the Questionnaire of Olfactory Disorders—Negative Statements (sQOD-NS)						
0–21	21	21	21	21	21	19
Quality of Life after Brain Injury questionnaire (QOLIBRI)						
0–100	80	93	78	77	80	86
Health-related quality of life (SF-36)						
Physical functioning 0–100	**75**	95	**60**	**75**	**80**	**75**
Physical role functioning 0–100	100	100	100	**0**	100	**0**
Bodily pain 0–100	100	100	78	78	80	100
General health perceptions 0–100	75	70	70	*65*	**60**	90
Vitality 0–100	**40**	85	*55*	*55*	60	80
Social role functioning 0–100	*75*	100	100	**63**	100	*75*
Emotional role functioning 0–100	**33**	100	100	100	**33**	100
Mental health 0–100	72	76	80	84	68	92
The Post-COVID-19 Functional Status (PCFS) scale						
Before (0–4)	0	0	0	0	1	1
After (0–4)	1	1	1	2	2	2

### Main symptoms during the acute and/or postacute stage

One patient (P1) suffered from Guillain–Barré syndrome and the other five (P2–P6) from severe ARDS; two patients (P1 and P2) presented with ICU delirium. All six patients presented with ventilator-associated pneumonia as well as other COVID-19-related complications (P1: hypernatraemia; P2: hypoxaemia and hypernatraemia; P3: hepatitis and renal insufficiency; P4: hypoxaemia, pulmonary embolism, septic shock, and hypernatraemia; P5: hypoxaemia, pulmonary superinfection, pulmonary embolism, and hypernatraemia; P6: hypoxaemia, septic shock, and renal insufficiency). Structural 3T MRI examination was performed during the acute stage in five patients (P1 and P3–P6). Along with qualitative analysis, automated morphometric segmentation of a T1-weighted image, magnetization-prepared rapid gradient echo (MPRAGE) sequence was done with the MorphoBox software (Siemens, Erlangen, Germany), which includes the comparison with a population of 303 healthy age and sex-matched control subjects [24]. This quantitative analysis was normal in all of the five patients; one patient (P4) presented signs of endothelial microlesions in the splenium of the corpus callosum.

### Medical history

Three patients had known risk factors (P2: hypertension, obesity, and hypercholesterolaemia; P3: hypertension and obstructive sleep apnea syndrome; P6: obstructive sleep apnea syndrome and obesity). None of the patients had recent surgical interventions.

### Postacute follow-up

One patient returned directly from the acute ward home, whereas the other five benefited from postacute inpatient rehabilitation, which lasted between 14 and 53 days (Table 1).

A total of 8 of the 12 patients, who were removed from the one-year follow-up (see above), have granted the CHUV general permission to use their clinical data for research. We have compared their characteristics with those of the six patients who participated in this study. Between these two groups, there was no significant difference demonstrated with the Mann–Whitney test in age (*p* = 0.345), duration of acute inpatient stay (*p* = 0.662), duration of ICU stay (*p* = 1.000), or duration of mechanical ventilation (*p* = 0.755). Furthermore, there was no significant difference in education level (*p* = 0.165), presence of ARDS (*p* = 0.429), or occurrence of delirium (*p* = 0.627), as demonstrated using the exact Fisher test.

All patients underwent a brief neuropsychological examination at 9 months (not reported here in detail), which revealed preserved visual field (National Institute of Health Stroke Scale of 0/3), preserved visuospatial attention (assessed by means of visual extinction with double simultaneous stimulation, clock drawing, scene copy, overlapping figures, two line-bisection, and Bell’s cancellation) [25] as well as preserved verbal and nonverbal episodic memory (assessed with the RL/RI-16 free and cued recall task [26] and the delayed recall of the Taylor complex figure) [27]. The apparent absence of episodic memory and lateralized attention deficits made us confident to use self-report questionnaires and visual analogue scales during the evaluation at 12 months post-COVID-19.

At 12 months after the onset of COVID-19, all patients had returned to work, although two (P4 and P6) returned only part-time and three (P1, P3, and P5) returned with schedules and tasks that needed to be adapted. All reported lesser efficiency in their professional activity, which they attributed to persistent fatigue and weakness, increased need for rest, and/or higher level of work-related stress.

In summary, patients included in this study had a detailed cognitive assessment at three time points following the diagnosis of COVID-19 (T0): (1) at the postcritical acute stage (that is, after leaving the ICU; T1), which is not reported here; (2) at 9 months (T2), which is briefly described above; and (3) at 12 months (T3), reported in detail here.

The study was approved by the Cantonal Ethics Committee of Vaud (Project Coro-Neuro 2020–01123). All patients, who participated in this study signed the informed consent, as stipulated by the requirements of the Ethics Committee, hereby agreeing to participate in the different parts of this study, to give the researchers access to their clinical data for the purpose of this study, and to grant them permission to transfer their coded data for research, including for publication purposes.

## Assessment

The cognitive assessment reported here involved 3 hours of testing, carried out on a single day, at the same time during the day (1–4 pm), by the same neuropsychologist, and in the same environment. The order in which scales and cognitive tests were administered is listed in Table 2. Two 10-minute breaks were inserted: the first after the first block of scales and questionnaires [that is, after the Post-COVID-19 Functional Status (PCFS) scale] and the second before the second block of scales [that is, before the Hospital Anxiety and Depression (HAD) scale; Table 2]. All patients reported sufficient sleep (7–8 hours/night) during the two nights preceding the assessment. None of the patients was under medication at the time of testing.

**Table 2:**
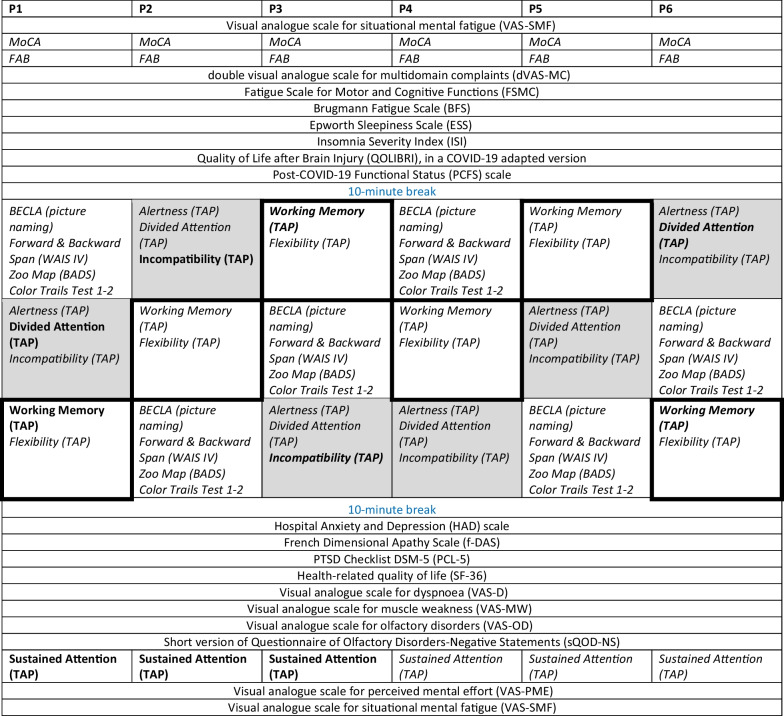
Order in which questionnaires, scales, and cognitive tests were administered in individual patients and level of cognitive performance in the latter (deficient performance in bold, the lower limit of the norm in bold–italics, and normal in italics). Three blocks of tests were pseudorandomized across subjects (individual blocks are indicated by white or gray background or by black edge, respectively). Note that three tests were administered in the same order for all patients, two at the beginning (MoCA and FAB) and one at the end of the session (sustained attention, TAP)

### Neuropsychological assessment

Neuropsychological assessment comprised two standardized test batteries: the Montreal Cognitive Assessment (MoCA; https://www.mocatest.org) and the Frontal Assessment Battery (FAB; www.psychdb.com/cognitive-testing/fab). Additional tests included the Batterie d’Évaluation Cognitive du Langage (BECLA) naming test [28], the forward and backward spans (WAIS IV) [29], the Zoo Map Test [30], the Color Trails Test [31] as well as the TAP subtests for alertness, divided attention, flexibility, incompatibility, working memory, and sustained attention [32]. For each patient the level of performance was compared with the norms of each test and is described here as severely deficient (< 2nd percentile), moderately deficient (≥ 2nd and ≤ 5th percentile), at the lower limit of normal performance (> 5th and < 16th percentile), or normal (≥ 16th percentile).

### Fatigue and fatigability

We assessed perception of fatigue and fatigability in two ways, respectively. First, as a subjective perception of fatigue by means of four self-report scales (as described below), including chronic characteristics (trait perceptions) of fatigue and of rest propensity and momentary (state) perceptions of mental fatigue and of perceived mental effort [33–36], perception of effort being a factor which contributes to the perception of fatigue [34]. Second, as a subjective assessment of mental fatigability by quantifying the increased level of self-reported mental fatigue between two time points [37] and as an objective assessment of mental/cognitive fatigability by quantifying the decline in one or more aspects of performance during continuous performance of a prolonged task [34, 38, 39].

Self-reported fatigue was assessed with two standardized scales and two visual analogue scales (Table 1). For each scale, higher scores indicated greater fatigue.

1. The Fatigue Scale for Motor and Cognitive Functions (FSMC) [40] corresponds to chronic characteristics (trait self-perceptions) of fatigue, or “day-to-day” fatigue, comprising ten items for motor and ten items for cognitive fatigue, with each item measured on a five-point Likert scale (1–5)
2. The Brugmann Fatigue Scale (BFS) [41] corresponds to chronic characteristics (trait self-perceptions) of rest propensity, comprising four items for physical and four for mental fatigue, with special focus on propensity to rest, with each item measured on a four-point Likert scale (0–3)
3. The visual analogue scale for situational mental fatigue (VAS-SMF) corresponds to instantaneous (state) self-perceptions of fatigue, or fatigue “in-the-moment”, with which each patient estimated on a 10-cm scale the level of mental fatigue at two given points: before versus after completing the neuropsychological assessment
4. The visual analogue scale for perceived mental effort (VAS-PME), with which each patient indicated on a 10-cm scale the level of mental effort they experienced during the neuropsychological assessment.

Mental/cognitive fatigability was measured by calculating the change of situational mental fatigue (VAS-SMF), self-reported before and after completing the neuropsychological assessment and by quantifying the objective decline of performance during a sustained mental effort rated by a sustained attention task, which lasted 15 minutes divided into three intervals of 5 minutes and was administered at the end of the assessment.

### Further specialized scales

Specialized scales were used as follows: anxiety and depression were assessed with the Hospital Anxiety and Depression (HAD) scale [42]; apathy was assessed with the French Dimensional Apathy Scale (f-DAS) [43]; post-traumatic stress disorder (PTSD) was assessed with the DSM-5 PTSD checklist (PCL-5) [44]; sleepiness was assessed with the Epworth Sleepiness Scale (ESS) [45]; insomnia was assessed with the Insomnia Severity Index (ISI) [46]; dyspnoea was assessed with the visual analogue scale for dyspnoea (VAS-D); muscle weakness was assessed with the visual analogue scale for muscle weakness (VAS-MW); olfactory disorders were assessed with the visual analogue scale for olfactory disorders (VAS-OD) and the short version of Questionnaire of Olfactory Disorders—Negative Statements (sQOD-NS) [47]. For the assessment of the quality of life, the questionnaire Quality of Life after Brain Injury (QOLIBRI) [48] was used in a COVID-19-adapted version (three items, which referred specifically to traumatic brain injury, were reformulated); for the general health status, the health-related quality of life (SF-36) assessment was used [49]; and for the functional status, the Post-COVID-19 Functional Status (PCFS) scale [50].

**Fig. 1:**
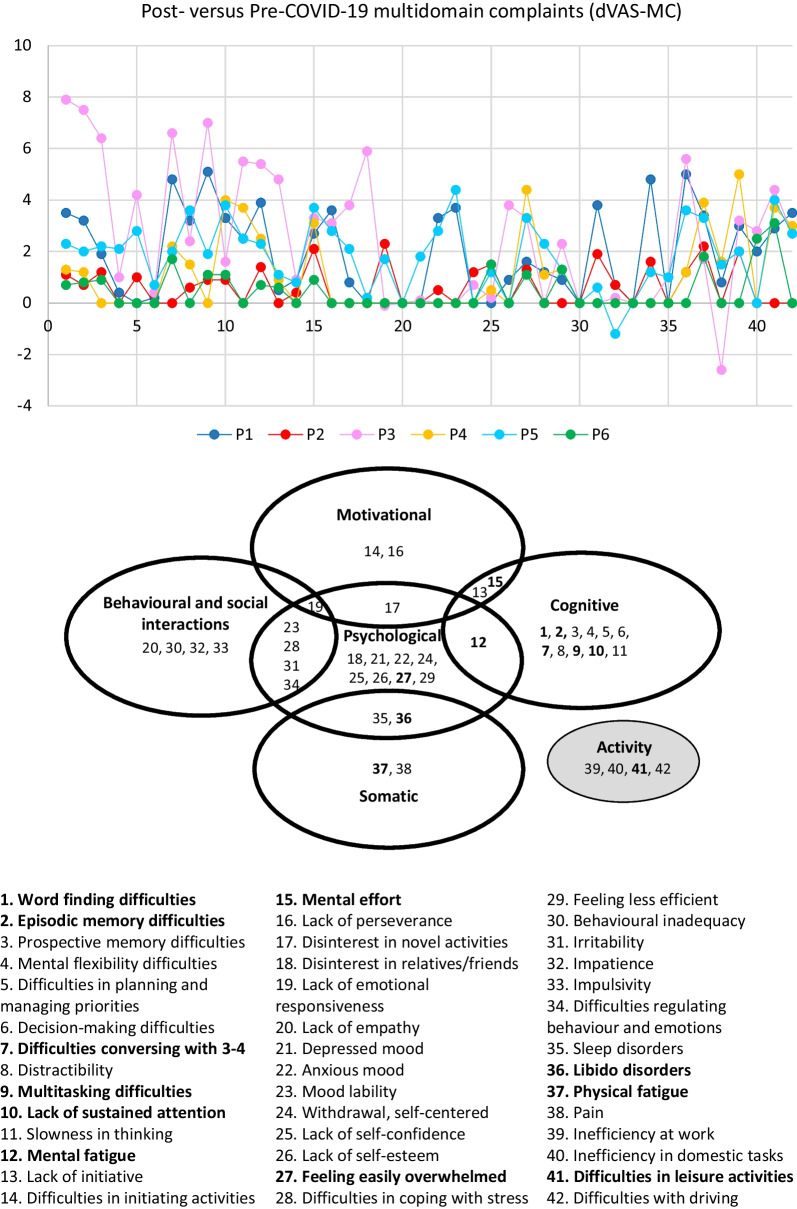
COVID-19-related changes in multidomain complaints as assessed by the double visual analogue scale for multidomain complaints (dVAS-MC). Top panel: changes in complaints as reported by patients P1–P6. Scale items (as listed below) are indicated on the *x*-axis and post- versus pre-COVID-19 ratings on the *y*-axis. Bottom panel: attribution of the scale items to cognitive, motivational, behavioral and social interactions, psychological and/or somatic domains, or different types of activity (highlighted in gray). Items, in which post- minus predifference were greater than the minimal important difference (MID), are in bold

Pre- versus post-COVID-19 complaints were compared by means of the double visual analogue scale for multidomain complaints (dVAS-MC). This scale was developed to cover the different domains, which are typically affected in the aftermath of severe COVID-19 [17], of other coronavirus infections, or of ARDS of other etiologies [20, 51, 52]. The scale includes 38 items, which cover (1) cognition, (2) motivation, (3) behavior and social interactions (4) psychological, and (5) somatic dysfunction (Fig. 1). In addition, the scale comprises functional appreciation of performance in four activities: (1) work, (2) domestic tasks, (3) leisure, and (4) driving. Each patient indicated for each of the 38 items the level of their complaints and, for each type of activity, their performance level as perceived before COVID-19 and at the time of testing (that is, at 12 months post-COVID-19 onset); the difference between the latter and the former score characterizes the impact of COVID-19.

### Data analysis

Performance of individual patients in the above listed psychometric tests and self-reported scales are listed in Tables 1, 2, 3 or illustrated in Fig. 1. Normal performance in standardized tests is as defined in the relevant publications, also cited above.

**Table 3:** Raw scores in psychometric tests of patients (P1–P6) at the top of the table: normal performances in italics. The percentiles in the subtests of the TAP (P1–P6) at the bottom of the table: deficient performance (severely deficient < 2nd percentile and moderately deficient ≥ 2nd and ≤ 5th percentile), performance at the lower limit of the norm (> 5th and < 16th percentile), and normal performance (≥ 16th percentile)

	P1	P2	P3	P4	P5	P6
*Raw scores in psychometric tests*						
MoCA	*30*	*29*	*29*	*30*	*27*	*30*
FAB	*18*	*17*	*18*	*18*	*18*	*17*
BECLA (picture naming)	*20*	*20*	*20*	*20*	*20*	*20*
Forward span (WAIS IV)	*6*	*6*	*5*	*6*	*6*	*5*
Backward span (WAIS IV)	*4*	*5*	*4*	*5*	*4*	*4*
Zoo map (BADS) Profile	*4*	*4*	*4*	*4*	*4*	*4*
Color Trails Test 1						
Time (seconds)/errors	*37/0*	*42/0*	*48/0*	*51/0*	*53/0*	*56/0*
Color Trails Test 2						
Time (seconds)/errors	*78/0*	*87/0*	*86/0*	*92/0*	*96/0*	*109/0*
Interference index	*1.1*	*1.1*	*0.8*	*0.8*	*0.8*	*0.9*
*Percentiles in the subtests of the TAP*						
Alertness (TAP)						
Median RT (Tonic/Phasic)	80/82	84/86	79/80	98/97	98/99	82/84
SD (Tonic/Phasic)	90/93	97/93	82/66	84/86	97/98	76/79
Divided attention (TAP)						
Median RT (Auditory/Visual)	5/92	31/73	50/82	82/93	18/21	10/93
SD (Auditory/Visual)	69/96	21/92	16/62	18/79	38/76	50/66
Omissions	84	34	86	86	58	38
Working memory (TAP)						
Median RT	34	93	18	24	84	86
SD	5	97	21	21	79	14
Errors/Omissions	73/96	50/97	10/24	73/98	73/95	> 76/99
Flexibility (TAP)						
Median RT	84	31	24	96	98	96
SD	86	42	24	96	96	90
Errors	> 86	31	> 86	84	84	84
Incompatibility (TAP)						
Median RT	58	4	16	82	79	79
SD	58	21	8	97	98	58
Errors	73	5	99	73	100	< 99
Sustained Attention, form (TAP)						
Median RT (0–5/5–10/10–15)	27/31/16	18/16/12	18/21/27	58/84/82	31/50/50	50/58/66
SD (0–5/5–10/10–15)	76/58/4	14/14/16	46/50/10	46/54/76	73/66/73	92/73/62
Errors (0–5/5–10/10–15)	> 66/ > 46/ > 42	> 66/ > 46/38	31/4/1	31/ > 46/ > 42	> 66/ > 46/ > 42	> 66/ > 46/ > 42
Omissions (0-5/5–10/10–15)	> 58/58/ > 54	8/18/4	54/ > 66/50	> 58/58/ > 54	21/24/50	> 58/ > 66/ > 54

BADS, Behavioral Assessment of the Dysexecutive Syndrome; BECLA, Batterie Cognitive d'Évaluation du Langage; FAB, Frontal Assessment Battery; MoCA, Montreal Cognitive Assessment; RT, reaction time; SD, standard deviation; TAP, Test for Attentional Performance

As for fatigue and fatigability [34], complaints in other domains tend to occur in normal subjects. We have therefore defined clinical significance as follows: the most prominent differences in pre- versus post-COVID-19 complaints, assessed by means of the double visual analogue scale for multidomain complaints (dVAS-MC), were identified in three steps. First, for each patient, the scales were ordered according to the post- minus prescore. The highest weight (42) was given to the scale with the highest post- minus predifference; the scale with the second largest difference received a weight of 41 and so on, until the scale with the lowest post- minus predifference received a weight of 1. Second, for each scale, the weights obtained for the six patients were summed and the scales reordered according to these sums. The first scale corresponded to the scale with the largest post- minus predifference across the patients; the second scale had the second largest difference and so on, until the last scale that had the smallest post-minus predifference. Thirdly, the scales with the 25% largest differences were further kept, that is, corresponding to 11 scales among the 42. These 11 items are most likely clinically relevant, since the post-minus predifference was greater than the minimal important difference (MID), defined in previous publications, at ≥ 1.5 [53].

For specific hypotheses, correlations between scales have been analyzed using Spearman correlations; the Rho and *p* values are reported when significant.

## Results

### Performance in standardized neuropsychological tests

At 12 months post-COVID-19 onset, all six patients obtained scores within normal limits in the two cognitive batteries MoCA and FAB (Tables 2 and 3). Additional tests confirmed good performance in several cognitive domains, such as picture naming (BECLA), forward and backward memory span (WAIS IV), executive functions (Zoo Map Test, Color Trails Test 2, and Flexibility TAP), as well as alertness (TAP) and processing speed (Color Trails Test 1).

A total of four patients were deficient or at the lower limit of normal performance in at least two of the working memory, executive, or attentional tests: P1 in divided attention, working memory, and sustained attention; P2 in incompatibility and sustained attention; P3 in working memory, incompatibility, and sustained attention; and P6 in divided attention and working memory. Three patients (P1, P2, and P3) presented a decrease in performance during the three time intervals of the sustained attention task (used as a measure of mental/cognitive fatigability) administered at the end of the exam, in terms of median reaction times, fluctuating reaction times, errors, or omissions (Tables 2 and 3). It is to be noted that deficient or low performance at tests of working memory, incompatibility, and divided attention was present when these tests were administered at the beginning (P2, P3, P5, and P6) or at the end (P1, P3, P4, and P6) of the neuropsychological assessment, independent of the order in which the test were administered (Table 2).

### Level of fatigue and fatigability

All six patients indicated chronic characteristics (trait self-perceptions) of fatigue, or “day-to-day” fatigue (Table 1). Indeed, the Fatigue Scale for Motor and Cognitive Functions (FSMC) [40] highlighted fatigue in all six patients. The motor subscale revealed the presence of severe fatigue in two patients (P4 and P5), moderate fatigue in two others (P1 and P3), and mild fatigue in two others (P2 and P6); the cognitive subscale revealed the presence of moderate fatigue in four patients (P1, P3, P4, and P5) and mild fatigue in two others (P2 and P6). Within the Brugmann Fatigue Scale (BFS), which assesses chronic characteristics (trait self-perceptions) of rest propensity [41], the physical subscale revealed moderately increased propensity in four patients (P1, P3, P4, and P5), and the mental subscale revealed moderately increased propensity in three patients (P1, P3, and P5).

Considering instantaneous (state) self-perceptions of fatigue, or fatigue “in-the-moment”, assessed by means of the VAS-SMF (Table 1), all six patients reported an increased level of mental fatigue after, as compared with before, neuropsychological evaluation, and for five of them (P1–P5), this increase was greater than the clinically relevant MID of 1.5 [53] as a subjective assessment of mental fatigability. The level of mental effort a patient experienced during the neuropsychological evaluation, which is a factor contributing to the instantaneous (state) self-perceptions of fatigue, was assessed with the VAS-PME (Table 1). The individual scores varied between patients (mean ± SD of 5.26 ± 2.35).

The four patients (P1, P3, P4, and P5) with the highest scores on the Fatigue Scale for Motor and Cognitive Functions (FSMC) [40] also reported need for rest (BFS) [41] and the highest level of perceived mental effort (VAS-PME) during the examination. They also presented increases in post- versus prelevels of situational mental fatigue (VAS-SMF) greater than the clinically relevant MID of 1.5 [53]. During the sustained attention test, unlike P4 and P5 who performed well, the response times of P1 fluctuated, and P3 presented high level of error during the last two intervals; these aspects are often interpreted as sign of mental/cognitive fatigability. The situational mental fatigue report for P2 post- versus pre-evaluation (VAS-SMF) increased by a factor of 2.4, which is well above MID of 1.5 [53], and sustained attention performance decreased (in terms of omissions) for the last time interval, while chronic self-perceptions of fatigue (FSMC) [40] was mild and need for rest (BFS) [41] was normal. Finally, for P6: the chronic self-perceptions of fatigue (FSMC) [40] were mild, need for rest (BFS) [41] was normal, VAS-PME was not high, situational mental fatigue report post-vs. pre-evaluation (VAS-SMF) was less than the clinically relevant MID of 1.5 [53], and sustained attention performance was normal; all the results were coherent with each other.

Scales assessing the chronic characteristics of cognitive fatigue (FSMC) [40] and the chronic characteristics of mental rest propensity (BFS) [41] correlated to each other (*R* = 0.953, *p* = 0.003). Moreover, the score of the VAS-PME, which assessed the level of mental effort the patient experienced during the neuropsychological assessment, was correlated with the mental rest propensity (BFS; *R* = 0.926, *p* = 0.008) and the cognitive fatigue (FSMC; *R* = 0.971, *p* = 0.001).

### Psychiatric, neurobehavioral, and somatic symptoms

Psychiatric and neurobehavioral symptoms were present in some patients (Table 1). The Hospital Anxiety and Depression (HAD) scale [42] identified one patient with a borderline anxiety score (P5), whereas the depression scores of all patients were within normal limits (Table 1). The French Dimensional Apathy Scale (f-DAS) [43] revealed abnormal scores for the executive dimension in two patients (P1 and P3), whereas the emotional and initiative dimensions were within normal limits for all patients. None of the patients presented symptoms of PTSD as documented by the DSM-5 PTSD checklist (PCL-5) [44].

Sleep was not a major problem in our population (Table 1). The Epworth Sleepiness Scale (ESS) did not reveal increased daytime sleepiness in any of the patients compared with healthy adults [45]. Two patients (P1 and P5) reported mild insomnia, and the other four reported no clinically significant insomnia on the Insomnia Severity Index (ISI) [46].

Typical post-COVID-19 somatic complaints were reported by several patients (Table 1). The visual analogue scale for dyspnoea (VAS-D) documented discomfort in four patients, with a 3/10 in two of them (P3 and P6) and a 7/10 in the other two (P4 and P5). A total of three patients (P4, P5, and P6) reported muscle weakness on the visual analogue scale for muscle weakness (VAS-MW), and one patient reported (P6) olfactory dysfunction on the visual analogue scale for olfactory disorders (VAS-OD), while scores were for all normal on the short version of Questionnaire of Olfactory Disorders—Negative Statements (sQOD-NS) [47].

The Quality of Life after Brain Injury questionnaire (QOLIBRI), in its adapted COVID-19 form, showed normal scores compared with healthy females/males in the same age range and educational level for all patients [54]. The health-related quality of life questionnaire (SF-36) revealed that, in comparison with a healthy volunteers group on the French version of the SF-36 health status questionnaire [49], five patients (P1, P3, P4, P5, and P6) had low scores in at least one domain and four (P1, P4, P5, and P6) in two or more domains. The Post-COVID-19 Functional Status (PCFS) scale [50] assesses functional status on an ordinal tool over time, before and after COVID-19. After COVID-19, all patients reported an increase in difficulties, which correspond to negligible (P1, P2, and P3) or slight functional limitations (P4, P5, P6).

Thus, 12 months after the onset of COVID-19, the negative impact of the disease was apparent in our population when using the SF-36 [49], the PCFS scale [50], the VAS-D, the VAS-MW, the VAS-OD, and the f-DAS [43]. In contrast, minor or no problems were reported with the HAD scale [42], the PCL-5 [44], the ESS [45], the ISI [46], the sQOD-NS [47], and the QOLIBRI, in its adapted COVID-19 form [48, 54].

### COVID-19-related multidomain complaints

Multidomain complaints present at 12 months after COVID-19, as compared to before, were assessed by means of the dVAS-MC, which comprises 38 items covering cognitive, motivational, behavioral and social interactions, psychological and somatic domains, as well as four functional characteristics. Longitudinal comparison, that is, before versus 12 months after COVID-19, revealed an overall increase in scores (Fig. 1). The average increase was calculated for each item. The 11 items with the largest difference in our case series were (in decreasing order): (1) physical fatigue, (2) mental effort, (3) mental fatigue, (4) feeling easily overwhelmed, (5) word-finding difficulties, (6) difficulties conversing with 3–4 (with more than 2 people), (7) lack of sustained attention, (8) multitasking difficulties, (9) difficulties in leisure activities, (10) libido disorders, and (11) episodic memory difficulties. The clinical relevance of these 11 items is furthermore warranted by the post- versus predifference, which was greater than the minimal important difference (MID), defined in previous publications at ≥ 1.5 [53].

Difficulties in several domains of the dVAS-MC correlated with the level of mental effort the patient experienced during the neuropsychological assessment, as assessed by the VAS-PME. This was the case for word-finding difficulties (*R* = 0.900, *p* = 0.037), episodic memory difficulties (*R* = 1.000, *p* < 0.001), multitasking difficulties (*R* = 0.829, *p* = 0.042), mental fatigue (*R* = 0.886, *p* = 0.019), and libido disorder (*R* = 0.899, *p* = 0.015).

**Fig. 2:**
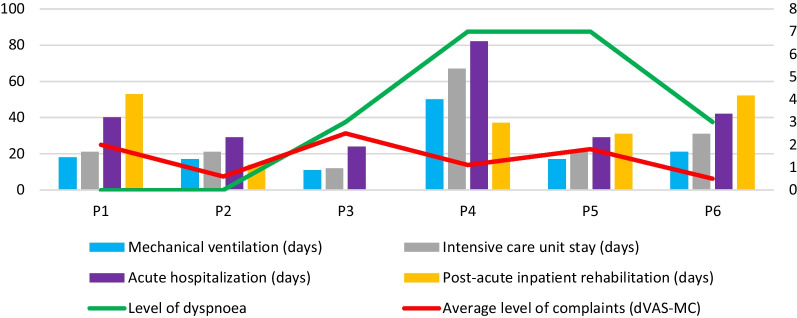
Average level of complaints as assessed by the dVAS-MC compared with clinical characteristics collected during the acute or postacute stages (duration of mechanical ventilation, intensive care unit stay, acute hospitalization, and postacute inpatient rehabilitation) or at the time of testing (level of dyspnoea). The left scale, in days, concerns acute and postacute characteristics (bars), the right scale indicates scores (1–10) collected at the time of testing (lines)

There was no apparent relation between the average level of complaints, as assessed by dVAS-MC, and the level of dyspnoea reported by patients or the duration of mechanical ventilation, ICU stay, acute hospitalization, and postacute inpatient rehabilitation (Fig. 2).

## Discussion

Our study provides a fine-grained evaluation of post-COVID-19 fatigue/fatigability, multidomain complaints, pattern of cognitive deficits, and neurobehavioral/psychiatric/somatic dysfunction in a series of patients who suffered from a severe form of the disease and who were treated in ICU.

A high prevalence of fatigue and cognitive deficits in convalescent COVID-19 and postcritical chronic illness has already been well established [21, 22]. Unlike in previous studies, however, none of our patients had a history of neurological or psychiatric disease or cognitive dysfunction and did not report prior fatigue symptoms. Thus, the COVID-19-related symptoms described here are not merely a worsening of a preexisting condition.

### Fatigue and fatigability

Previous publications highlighted the multidimensional nature of fatigue as well as the poor understanding of its mechanisms [55]. Clinically fatigue tends to be defined as “a subjective lack of physical and/or mental energy that is perceived by the individual or caregiver to interfere with usual and desired activities” [56]. In the context of neurologic diseases, fatigue is often defined as the subjective sensation, that is, reported by the patient, whereas the impact on performance in tests is referred to as fatigability [34]. There are also different means of measuring the perception of fatigue, including momentary (state) perceptions and chronic characteristics (trait perceptions). In this context, we sought to measure different aspects of fatigue (trait and state) while also distinguishing fatigue from fatigability.

Our case series highlights the prominence of chronic characteristics (trait perceptions) of fatigue and the scale used, namely the Fatigue Scale for Motor and Cognitive Functions (FSMC). Those with higher scores on this scale of fatigue (P1, P3, P4, and P5) also had a significant propensity to rest (trait perceptions), reported more mental effort (state perceptions), and a situational mental fatigue post- versus pre-evaluation (state perceptions) greater than the clinically relevant MID of 1.5 [53], which is interpreted as an indicator of mental fatigability [37]. Among those patients who have these consistent complaints on different aspects of fatigue (trait and state) and fatigability, two had normal cognitive performance (P4 and P5), while the other two (P1 and P3) presented cognitive dysfunction or performance at the lower limit of the norm in some, but not all, attentional, executive, and/or working memory tests and a decline of performance during a sustained mental effort rated by the sustained attention task administered at the end of the exam, interpreted as a mental/cognitive fatigability [34, 38, 39]. Conversely, cognitive dysfunction or performance at the lower limit of the norm in some, but not all, attentional, executive, and/or working memory tests were also noted in patients (P2 and P6) with a mild chronic characteristics (trait perceptions) of fatigue (FSMC). We also noted a decline of performance on the sustained attention task in P2. Thus, subjective complaints of fatigue [chronic characteristics (trait perceptions) of fatigue or of rest propensity and momentary (state) self-perceptions of fatigue and of mental effort] and cognitive complaints were accompanied for some (P1, P2, P3, and P6) but not for others (P4 and P5) of cognitive disorders on examination, confirming distinct profiles described in literature (as detailed in the next paragraph) between fatigue, fatigability, cognitive complaints, and cognitive disorders. Additionally, we can wonder about the bidirectionality of the link between cognition and fatigue. Both mechanisms could be at play, by fatigue worsening cognitive performance and/or the mental effort needed for a cognitive task triggering or accentuating fatigue. Related to this, it is also interesting to consider the profile of P4 and P5 who had normal cognitive performance but were among the three patients (P3, P4, and P5) who had the greatest increase of situational mental fatigue between the two time points (post- versus pre-evaluation) as a mental fatigability indicator and who also had cognitive complaints. This situational mental fatigue associated with cognitive complaints and preserved cognitive performance has previously been interpreted by efficient (but costly) compensatory processes [57].

### Fatigue, fatigability, cognitive complaints, and cognitive impairment

In our patient population, the pervading presence of mental and physical fatigue and of cognitive-related complaints (mental effort, mental fatigue, feeling easily overwhelmed, word finding difficulties, difficulties conversing with 3–4 (with more than 2 people), lack of sustained attention, multitasking difficulties, and episodic memory difficulties) were accompanied by mental fatigability and by deficits in some, but not all, attentional, executive, and/or working memory tests; mental fatigability was found in five patients (P1–P5; Table 1), and deficient performance or performance at the lower limit of the norm in these tests was found in four patients (P1–P3 and P6; Table 2). An unsystematic or even poor correlation between questionnaires and formal testing is not new and has been reported in previous studies [58]; self-ratings reflect more closely the subjective experience of difficulties in everyday life and/or a possible decrease in performance as compared with the previous level of functioning. This suggests that formal testing and subjective questionnaires are not interchangeable and should be used in combination to capture the whole range of difficulties faced by patients. Moreover, dissociation between subjective fatigue and normal cognitive performance is well documented in normal subjects, even in the context of prolonged testing; the excess of fatigue was proposed to be due to compensatory efforts, which are necessary to maintain an adequate level of performance [59]. Whether similar coping mechanisms are at play in patients after severe COVID-19 remains to be determined.

The relationship between self-reported fatigue and neuropsychological test performance has been investigated in the aftermath of stroke, traumatic brain injury, or multiple sclerosis. The association between self-reported fatigue and cognitive performance [36, 60–64] and the lack of it have been reported [65–68]. In the latter, however, patients experienced rapidly increasing fatigue and presented signs of increased distress [69] and cognitive complaints [70], resulting from the use of compensatory strategies, which require greater mental effort [57]. Fatigue-related deterioration of performance is mostly present in attentional, executive, and working memory tasks, which require effortful or controlled cognitive processing and, thus, exceed most likely available cognitive resources. This is precisely what we observed for four of our six patients. Mental fatigue was proposed to reflect imbalance between mental effort necessary for a task and the available neural resources [71]. Consistent with this interpretation, patients with multiple sclerosis tend to activate more extensively neural networks during the execution of cognitive tasks, possibly indicating that they require greater cerebral resources and effort than healthy subjects.

Chronic fatigue has been shown to be accompanied by changes in neural processing, as shown in electroencephalogram (EEG), magnetoencephalography (MEG), and positron emission tomography (PET) studies [72]. Acute fatigue paradigms, exploring the effect of brief sustained effort (mostly carried out in normal subjects), have shown that mental fatigue tends to impact different aspects of attention and activities such as driving [73, 74] to aggravate physical fatigue [75] and to impair physical performance [76]. Conversely, physical activities can affect mental fatigue [77]. Neural mechanisms involved in COVID-19-related chronic fatigue need to be further investigated.

### Outcome of severe COVID-19

A recent publication from Wuhan, China, reported 1-year cognitive outcome of over 3000 COVID-19 survivors who had no prior neurological disorders or family history of dementia [78]. A brief neuropsychological evaluation was carried out with the Telephone Interview for Cognitive Status. The authors found signs of cognitive decline more frequently in patients who suffered from severe, rather than nonsevere, COVID-19, that is, in patients who had history of ICU stay and mechanical ventilation. Similarly to this large-scale study, our fine-grained evaluation of patients who suffered from severe COVID-19 revealed at 1-year follow-up that the occurrence was relatively isolated in cognitive dysfunction or performance at the lower limit of the norm for four of the six patients in some, but not all, attentional, executive, and/or working memory tests. In addition, our study also provided information about the occurrence of pervading mental and physical fatigue as well as numerous multidomain complaints (including cognitive/functional complaints) and, for some patients, the occurence of mental fatigability, a certain degree of neurobehavioral (apathy) and/or psychiatric (anxiety) and/or somatic (dyspnoea, muscle weakness, olfactory disorders and/or minor sleep problems) dysfunction. These relevant data can be missed in large-scale studies that use less detailed assessments.

As highlighted from the very early stages of the COVID-19 pandemic, patients with severe form, especially after an ICU stay, necessitate multidisciplinary rehabilitation [51, 79, 80], which was the case for five of the six patients. Moreover, our patients presented lasting sequelae similar to those from previous coronaviruses [20]. Their situation very much resembles that of patients who sustained critical illness of other etiologies and who necessitated intensive care [52, 81–83]. Functional limitations that were present after, but not before, COVID-19 notably include difficulties in leisure activities (dVAS-MC) and lower efficiency in their professional activity, which all six patients attributed to persistent fatigue and weakness, increased need for rest, and/or higher level of work-related stress (anamnestic data) as well as poorer health-related quality of life, which, as described in our patient population, are consistent with previous studies that have described reduced quality of life in survivors of critical illness [82, 84–86].

### Next steps

We describe here post-COVID-19 fatigue/fatigability, multidomain complaints, pattern of cognitive deficits, and neurobehavioral/psychiatric/somatic dysfunction that occurred in patients who were infected during the first COVID-19 wave. This syndrome occurred without brain lesions, which could be detected on structural MRI. Previous studies pointed out that the postintensive care syndrome of the first wave resembled that observed after MERS and after critical illness of other etiologies, without, however, separating cases with versus without brain damage [10, 13]. Further comparisons between survivors of critical illness due to different SARS-CoV-2 variants or other etiologies would need to take into account the presence and extent of brain damage.

Our case series provides a fine-grained evaluation of post-COVID-19 multidomain symptoms. It is valid as pilot study for subsequent large scale clinical and/or imaging studies. Patients included in this study had a relatively high level of education, held gainful employment, and were socially well integrated. How representative they are of the whole population needs to be determined in further, large-scale studies. In addition to large-scale studies, further investigation into the neural basis of the fatigue syndrome associated with severe COVID-19 needs to be carried out with specific functional MRI paradigms to understand the neural mechanisms underlying the presence of fatigue/fatigability and multidomain complaints. The comparison with mechanisms involved in stroke recovery, such as the loss of specificity of specialized processing networks, may be of great interest [87, 88]. Furthermore, we need to have a better understanding of the fatigue syndrome that these patients presented to tailor appropriate outpatient rehabilitation programs. The effect of interdisciplinary rehabilitation programs combining cognitive, neurobehavioral, psychiatric, and somatic approaches needs to be evaluated. Providing targeted treatments for fatigue has the potential to effectively enhance both psychological wellbeing and quality of life, with the value, especially of nonpharmacological interventions, for fatigue already demonstrated [89–91].

## Conclusion and clinical message

Our case series illustrates that fatigue, fatigability, multidomain complaints (including cognitive/functional complaints, which to some extend to everyday life), cognitive dysfunction, or performance at the lower limit of the norm and a certain degree of neurobehavioral and/or psychiatric and/or somatic dysfunction can occur in the aftermath of severe COVID-19 and continue to persist at 12 months, even in the absence of neurological antecedents or of COVID-19-related stroke and/or cardiac arrest. Based on these results, we recommend to include subjective trait and state fatigue, as well as neurobehavioral/psychiatric/somatic and multidomain complaints, in post-COVID-19 assessment scales. More extensive and focused neuropsychological investigations, including, in particular, but not limited to, the objective measure of mental/cognitive fatigability, should be carried out whenever possible.

## Data Availability

https://zenodo.org/record/7043766

## Abbreviations:

ARDS: Acute respiratory distress syndrome
FAB: Frontal Assessment Battery
ICU: Intensive care unit
MID: Minimal important difference
MoCA: Montreal Cognitive Assessment
TAP: Test for Attentional Performance
